# Normal karyotype in myelofibrosis: is prognostic integrity affected by the number of metaphases analyzed?

**DOI:** 10.1038/s41408-017-0046-3

**Published:** 2018-01-12

**Authors:** Maura Nicolosi, Mythri Mudireddy, Naseema Gangat, Animesh Pardanani, Curtis A. Hanson, Rhett P. Ketterling, Ayalew Tefferi

**Affiliations:** 1 Departments of Internal Medicine and Laboratory Medicine, Mayo Clinic, Rochester, MN USA; 2 Departments of Internal Medicine and Laboratory Medicine, Mayo Clinic, Rochester, MN USA

## Correspondence

Primary myelofibrosis (PMF) is a clonal myeloproliferative neoplasm (MPN) characterized by anemia, marked splenomegaly, extramedullary hematopoiesis, profound constitutional symptoms, and a propensity to progress into acute leukemia, resulting in premature death^1^. These features in PMF are accompanied by three mutually exclusive driver mutations: *JAK2, CALR*, and *MPL*^2, 3^. Current prognostic systems in PMF are mostly based on clinical parameters, with the exception of the dynamic international prognostic system (DIPSS)-plus, which includes cytogenetic information^4^. DIPSS-plus classifies karyotype in PMF as being either “favorable” or “unfavorable”. The former includes normal karyotype or sole abnormalities of trisomy 9, del(13q), del(20q), translocation/duplication of chromosome 1 and loss of Y chromosome, while the latter includes all other abnormalities^5^. It is currently unknown whether or not the prognostic integrity of “normal” karyotype in PMF is affected by the number of metaphases examined, or the presence of single-metaphase abnormalities classically associated with myeloid disorders that do not otherwise meet the International System for Human Cytogenetic Nomenclature (ISCN) criteria for constituting “clonal” changes^6^. The current study addresses these issues in a consecutive series of 604 patients with PMF and “normal” karyotype.

The current study was approved by the institutional review board of Mayo Clinic (Rochester, MN). Study patients were recruited from the institutional database of MPN. Diagnosis of PMF was according to the World Health Organization criteria^1^. Clinical and laboratory data, and cytogenetic information were collected at the time of diagnosis. Cytogenetic analysis and reporting were done according to the ISCN^6^. Fresh bone marrow aspirates were processed according to standard techniques using GTL banding with trypsin and Leishman stain^7^. Chromosomal abnormalities were considered “clonal” if the same structural abnormality or extra chromosome appeared in at least two and monosomy in at least three metaphases. Driver mutation screening and targeted next generation sequencing were performed as previously described^8, 9^. Differences in the distribution of continuous variables between categories were analyzed by Mann–Whitney or Kruskal–Wallis test. Patient groups with nominal variables were compared by *χ*^2^ test. Overall survival (OS) was considered from the date of diagnosis or referral to the date of death (uncensored) or last follow-up (censored). Leukemia-free survival (LFS) was calculated from the time of diagnosis or referral to the time of leukemic transformation (uncensored) or last contact or date of death (censored). Survival curves were prepared by the Kaplan–Meier methods and compared by the log–rank test. Cox proportional hazard regression model was applied for multivariable analysis. *P*-value <0.05 was considered significant. The Stat View (SAS Institute, Cary, NC, USA) statistical package was used for all calculations.

A total of 604 PMF patients with normal karyotype constituted the current study population. Clinical and laboratory characteristics at time of diagnosis or referral are listed in Table 1. Median age of the study patients was 65 years and 62% were males. Two hundred and seven (34%) patients were red cell transfusion dependent at time of initial evaluation, with median values of hemoglobin, leukocytes and platelets at 10.2 g/dl, 9 × 10^9^/l, and 242 × 10^9^/l, respectively. Constitutional symptoms were documented in 32% of patients and circulating blasts ≥1% in 50%. DIPSS^10^ and DIPSS-plus risk stratification were 9 and 30 high, 41 and 40% intermediate-2, 35 and 16% intermediate-1, and 15 and 14% low, respectively. Driver mutation analysis was available in 389 patients and included 250 (64%) patients with *JAK2*, 61 (16%) type 1/like *CALR*, 16 (4%) type 2/like *CALR*, 21 (5%) *MPL,* and 41 (11%) triple-negative mutational status. In addition, a subset of patients were screened for mutations in *ASXL1* (*n* = 245; 41% mutated), and *SRSF2* (*n = *256; 15% mutated).

**Table 1:** Clinical and laboratory characteristics of 604 primary myelofibrosis patients with normal karyotype, stratified by the number of metaphases studied or the presence of single-metaphase abnormalities classically associated with myeloid disorders that do not qualify as a “clone”, per ISCN criteria

Variables	All patients	Patients with presence of single-metaphase abnormality	Patients with normal karyotype and less than 10 metaphases evaluated	Patients with normal karyotype and 10–19 metaphases evaluated	Patients with normal karyotype and 20 or more metaphases	*p-* value
	(*n* = 604)	(*n* = 18) (3%)	(*n* = 51) (8%)	(*n* = 84) (14%)	(*n* = 451) (75%)	
Age in years; median (range)	65 (19–89)	65 (36–86)	67 (34–78)	63 (26–82)	65 (19–89)	0.2
Age ≥65 years; *n* (%)	308 (51%)	9 (50%)	31 (61%)	38 (45%)	230 (51%)	0.4
Sex (male); *n* (%)	374 (62%)	5 (28 %)	37 (73%)	59 (70%)	273 (61%)	**0.003**
Transfusion dependent; *n* (%)	207 (34%)	6 (33%)	21 (41%)	26 (31%)	154 (34%)	0.7
Hemoglobin, g/dL; median (range)	10.2 (5–16.1)	11.5 (7.2–16.1)	10 (6–16)	10.6 (5.8–16)	10.2 (5–16.1)	0.2
Hemoglobin <10 g/dL; *n* (%)	287 (48%)	8 (44%)	27 (53%)	33 (39%)	219 (49%)	0.4
Platelets, ×10^9^/L; median (range)	242 (8–2466)	185 (23–1754)	187 (11–818)	271 (11–1193)	245 (8–2466)	0.3
Platelets <100 × 10^9^/L; *n* (%)	122 (20%)	5 (28%)	14 (27%)	14 (17%)	89 (20%)	0.4
Leukocytes, ×10^9^/L; median (range)	9 (1–236.1)	10.3 (3–105.9)	6.6 (1-41)	7.7 (1.7–86)	10 (1–236.1)	**0.0004**
Leukocytes >10 × 10^9^/L; *n* (%)	274 (45%)	9 (50%)	13 (25%)	27 (32%)	225 (49%)	**0.0005**
Leukocytes >25 × 10^9^/L; *n* (%)	91 (15%)	2 (11%)	2 (4%)	12 (14%)	75 (17%)	0.1
Circulating blasts %; median (range)	0 (0–15)	0 (0–14)	0 (0–11)	0 (0–15)	1 (0–13)	0.7
Circulating blasts ≥1%; *n* (%)	301 (50%)	8 (44%)	22 (43%)	38 (45%)	233 (52%)	0.5
Constitutional symptoms; *n* (%)	191 (32%)	7 (39%)	14 (27%)	30 (36%)	140 (31%)	0.7
*DIPSS*						0.1
High; *n* (%)	55 (9%)	3 (17%)	1 (2%)	10 (12%)	41 (9%)	
Intermediate-2; *n* (%)	246 (41%)	6 (33%)	29 (56%)	26 (31%)	185 (41%)	
Intermediate-1; *n* (%)	214 (35%)	8 (44%)	15 (30%)	31 (37%)	160 (35%)	
Low; *n* (%)	89 (15%)	1 (6%)	6 (12%)	17 (20%)	65 (15%)	
*DIPSS-plus*						0.3
High; *n* (%)	181 (30%)	6 (33%)	13 (25%)	23 (27%)	139 (31%)	
Intermediate-2; *n* (%)	240 (40%)	7 (39%)	25 (49%)	25 (29%)	183 (41%)	
Intermediate-1; *n* (%)	96 (16%)	4 (22%)	7 (14%)	20 (24%)	65 (14%)	
Low; *n* (%)	87 (14%)	1 (6%)	6 (12%)	16 (20%)	64 (14%)	
Driver mutation “*N*” evaluable = 389 (64%)						0.7
*JAK2*; *n* (%)	250 (64%)	9 (64%)	15 (75%)	22 (58%)	204 (64%)	
Type 1 *CALR*; *n* (%)	61 (16%)	3 (22%)	3 (15%)	6 (16%)	49 (15%)	
Type 2 *CALR*; *n* (%)	16 (4%)	0 (0%)	0 (0%)	0 (0%)	16 (5%)	
*MPL*; *n* (%)	21 (5%)	1 (7%)	0 (0%)	3 (8%)	17 (6%)	
Triple negative; *n* (%)	41 (11%)	1 (7%)	2 (10%)	7 (18%)	31 (10%)	
*ASXL1*; *n* (%) “*N*” evaluable = 245 (41%)	101 (41%)	3 (38%)	4 (33%)	6 (25%)	88 (44%)	0.3
*SRSF2*; *n* (%) “*N*” evaluable = 256 (42%)	38 (15%)	1 (13%)	2 (17%)	2 (7%)	33 (16%)	0.7
Median follow-up in years; median (range)	3.5 (0–30.8)	3.8 (0–23.5)	3 (0–20.8)	3.6 (0–29.0)	3.5 (0–30.9)	0.5

The values in bold indicate a significant *p-*value (<0.05)

*DIPSS* dynamic international prognostic scoring system, *DIPSS-plus* dynamic international prognostic scoring system-plus, *JAK2* Janus kinase 2, *CALR* Calreticulin, *MPL* MPL proto-oncogene, *ASXL1* additional sex combs 1, *SRSF2* serine/arginine-rich splicing

The study population was stratified into four groups according to the number of metaphases studied and the presence or absence of the single-metaphase abnormalities classically associated with myeloid disorders that did not meet the ISCN criteria for clonal changes. The latter occurred in 18 (3%) patients, whereas among the remaining 586 cases, the number of metaphases studied was 20 or more in 451 (75%), 10–19 in 84 (14%), and <10 in 51 (8%). After a median follow-up of 3.5 years, 427 (71%) deaths and 40 (7%) leukemic transformations were documented. Phenotypic correlative studies disclosed no significant differences in the aforementioned four operational groups of “normal” karyotype, in terms of age (*p* = 0.2), red cell transfusion need (*p* = 0.7) hemoglobin level (*p* = 0.2), platelet count (*p* = 0.3), circulating blast count (*p* = 0.7), or constitutional symptoms (*p* = 0.7). DIPSS (*p* = 0.1) and DIPSS-plus risk distributions were also similar among the four groups (*p* = 0.3), as were driver mutational status (*p* = 0.7) and *ASXL1* (*p* = 0.3) and *SRSF2* (*p* = 0.7) mutational frequencies. The only difference of note was an association between leukocytosis and number of metaphases studied; the respective percentage of patients with ≥20, 10–19, <10 metaphases studied or with one abnormality were 82, 10, 5, and 3% in the presence and 68, 17, 12, and 3% in the absence of leukocytosis (*p* = 0.0005). In addition, we noted significantly more male patients with <10 metaphases analyzed (73 vs 61% for ≥20 metaphases, 70% for 10–19 metaphases and 28% for one abnormality; *p* = 0.003).

**Fig. 1:**
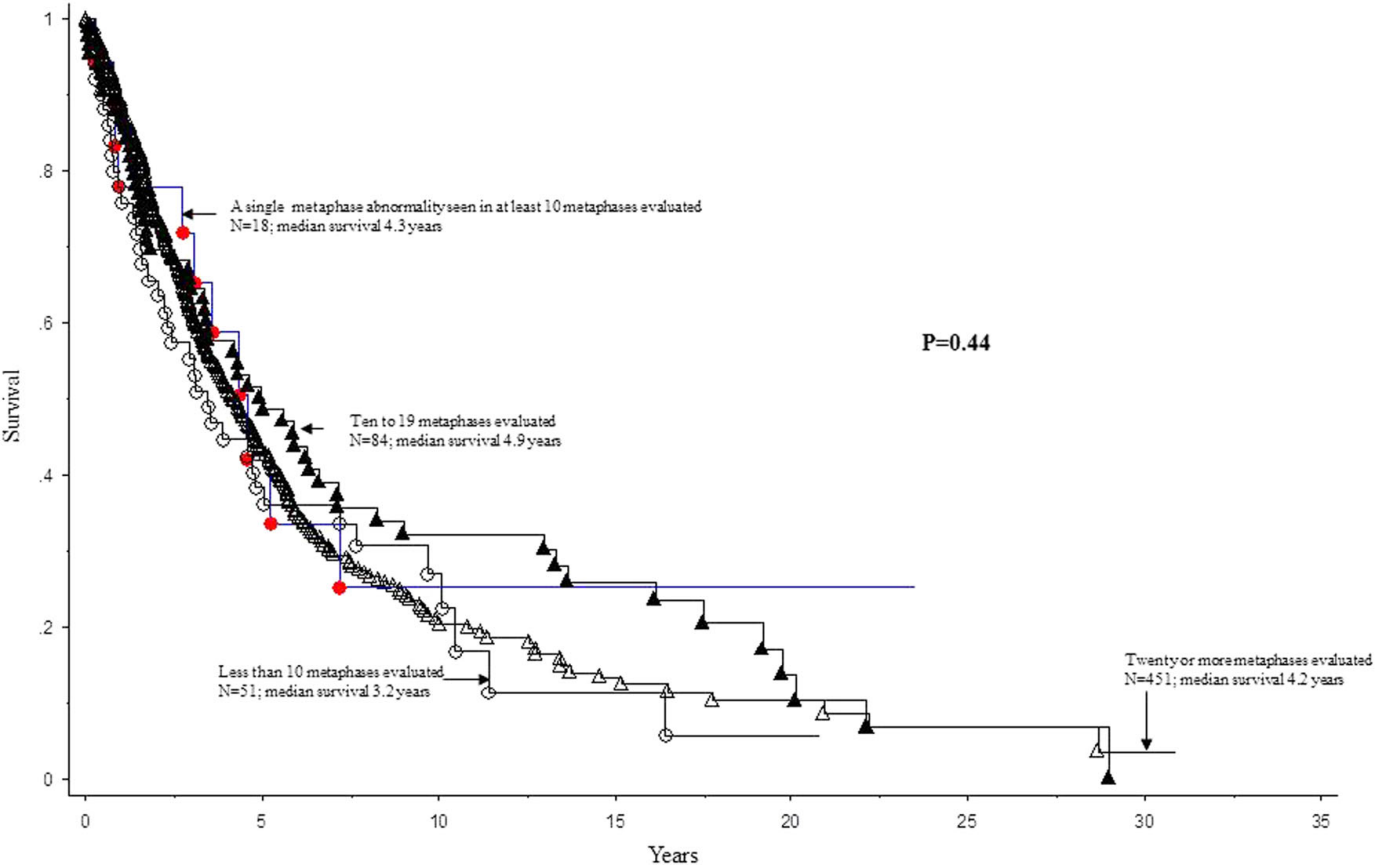
Survival of 604 patients with primary myelofibrosis and normal karyotype Survival in 604 patients with primary myelofibrosis and normal karyotype, stratified by number of metaphases evaluated and the presence of single-metaphase abnormalities classically associated with myeloid disorders that do not qualify as being “clonal” per ISCN criteria

In univariate analysis, all seven non-cytogenetic variables included in the DIPSS-plus risk model were significantly associated with shortened survival (*p* < 0.0001 for all instances). Significant risk factors in univariate analyses also included driver mutation status (*p* < 0.0001) and presence of *ASXL1* (*p* < 0.0001, HR 1.8; 95% CI 1.3–2.5) or *SRSF2* (*p* = 0.0001, HR 2.2; 95% CI 1.5–3.2) mutations. In univariate analyses LFS was adversely affected by hemoglobin <10 × 10^9^/L (*p* = 0.008, HR 2.4; 95% CI 1.3–4.5), circulating blasts count (*p* < 0.0001, HR 1.3; 95% CI 1.2–1.4) and presence of *SRSF2* mutation (*p* < 0.0001, HR 6.5; 95% CI 3–14.5). In contrast, neither OS nor LFS was affected by either the number of metaphases analyzed (*p* = 0.44; Fig. 1) or the presence of single-metaphase abnormalities classically associated with myeloid disorders that do not qualify as clonal changes (*p* = 0.42). This lack of prognostic impact from number of metaphases analyzed or presence of single abnormalities was confirmed by multivariable analysis that included age, gender, conventional risk stratification, or driver mutational status, as covariates.

The current study suggests that neither the number of metaphases studied nor the presence of single-metaphase abnormalities classically associated with myeloid disorders that do not qualify as clonal changes, per ISCN criteria, affect the prognostic integrity of a normal karyotype designation in PMF. Previous studies had cautioned that the number of analyzable metaphases from unstimulated blood and from bone marrow samples might be too few to confirm the absence of a cytogenetically detectable clone^11^. Consistent with this view, standard laboratory practice requires a minimum of 20 metaphases to be analyzed before reporting cytogenetic results out as “normal”. Other investigators generally agree on the need to examine at least 20 metaphases but suggested that full analysis of only 5 metaphases might be adequate^12^. In routine clinical practice, it is important for patients to be assured of the prognostic implication of a “normal” karyotype, particularly when this information is derived from the analysis of less than the standard 20 metaphases. Another practical implication of the current study concerns eligibility for a cytogenetic study inclusion of patients, in the absence of at least 20 or 10 metaphases analyzed. Regardless, we would like to underscore the fact that the current study was not designed to undermine the need for robust cytogenetic studies in PMF or other myeloid malignancies, but rather to provide a comparable statistical resource of prognostic integrity when a complete 20 metaphase chromosome study is not achievable.
